# Pathogenesis of Micro/Nanoplastics in Mammalian Systems: Gut to Systemic Multi-Organ Dysfunction

**DOI:** 10.34133/research.1439

**Published:** 2026-10-01

**Authors:** Zhimin Xu, Runtong Huang, Lei Wang, Yi Zhang, Asmaa A. Sharf-Eldin, Kui Xu, Mohamed G. Moussa, Renqiang Yu, Heru Pramono, Leli Wang, Yulong Yin

**Affiliations:** ^1^ Changsha Research Station for Agricultural & Environmental Monitoring, National Engineering Laboratory for Pollution Control and Waste Utilization in Livestock and Poultry Production, Institute of Subtropical Agriculture, Chinese Academy of Sciences, Changsha 410125, China.; ^2^ College of Environmental Science and Engineering, Nankai University, Tianjin 300350, China.; ^3^ The First Affiliated Hospital of Nanjing Medical University, Nanjing 210000, China.; ^4^ Department of Agricultural Botany, Faculty of Agriculture, Ain Shams University, Cairo 11566, Egypt.; ^5^ Department of Integrative Agriculture, College of Agriculture and Veterinary Medicine, United Arab Emirates University, Abu Dhabi, Al Ain 15551, United Arab Emirates.; ^6^ Department of Neonatology, Affiliated Women’s Hospital of Jiangnan University, Wuxi 214002, Jiangsu, China.; ^7^ Laboratory of Fisheries Microbiology, Department of Marine Science, Faculty of Fisheries and Marine, Universitas Airlangga, Surabaya 601115, Indonesia.

## Abstract

Micro- and nanoplastics (MNPs) have emerged as widespread environmental contaminants with growing implications for human health. Growing evidence indicates that the gastrointestinal tract is not only the primary site of exposure but also a central regulator of systemic toxicity through interactions among the intestinal barrier, immune system, and gut microbiota. Consequently, research has shifted from describing tissue accumulation to elucidating how intestinal dysfunction drives multi-organ effects via the gut–organ axis. This review summarizes recent advances in mechanisms by which ingested MNPs disrupt intestinal homeostasis and subsequently affect extraintestinal organs. We first examine how particle size, morphology, surface properties, environmental weathering, and polymer chemistry shape intestinal responses. Particular attention is given to the emerging view that biodegradable plastics, despite lower environmental persistence, may produce bioactive degradation products in the gastrointestinal tract that alter host–microbiota metabolism. We then integrate current evidence on how barrier disruption, microbial dysbiosis, altered microbial metabolites, and immune signaling mediate communication along the gut–liver, gut–brain, gut–lung, and gut–endocrine axes. In addition, we compare commonly used experimental models, highlighting their respective strengths and limitations for mechanistic studies and translational research. Finally, we discuss intervention strategies and key challenges, including environmentally realistic exposure assessment, methodological standardization, long-term biological adaptation, and the development of human-relevant models. Overall, MNP toxicity arises from dynamic interactions between particle properties and host responses rather than burden alone. Ultimately, MNP risk assessment should move beyond the “particle-accumulation” paradigm toward a “systemic-disruption” framework, where the gut is recognized as a biological amplifier of exposure and functional homeostasis takes precedence over particle burden.

## Introduction

Global plastic production now exceeds 400 million metric tons annually, and the resulting accumulation of plastic waste has led to the widespread presence of microplastics (MPs; 1 μm to 5 mm) and nanoplastics (NPs; <1 μm) throughout aquatic, terrestrial, and atmospheric environments [1,2]. Recent estimates indicate that 19 to 23 million metric tons of plastic waste enter aquatic ecosystems each year [3]. The burden may be even greater in terrestrial systems. Agricultural soils receiving wastewater sludge, reclaimed water, plastic mulch residues, and tire wear are estimated to contain micro- and nanoplastic (MNP) loads that are 4 to 23 times higher than those reported in marine environments [4,5]. As plastic contamination continues to accumulate across environmental compartments, exposure through food, drinking water, and airborne particles has become increasingly difficult to avoid.

Human exposure is now supported by a growing body of biomonitoring evidence. Although exposure estimates vary because of differences in analytical methods and particle-size detection limits, current assessments suggest that adults may ingest approximately 39,000 to 52,000 MNP particles annually through food consumption alone. When inhalation is included, total exposure may exceed 74,000 to 121,000 MNP particles per year [6,7].Drinking water represents an important source of exposure, with individuals relying primarily on bottled water estimated to ingest tens of thousands more MNP particles annually than those consuming tap water [8]. Synthetic polymer particles have been detected in human blood, placenta, breast milk, meconium, and other biological samples, indicating that environmental particles can cross biological barriers and reach internal tissues [9,10]. In one study, median MNP concentrations reached approximately 4,900 μg/g in frontal cortex samples, compared with roughly 433 and 404 μg/g in matched liver and kidney tissues, respectively [11,12]. These observations have shifted attention from environmental occurrence toward a more fundamental question: How do MNPs interact with biological systems after entering the body, and to what extent might these interactions contribute to human disease?

Although causal relationships remain difficult to establish, evidence linking MNP exposure with human health outcomes is rapidly accumulating. The gastrointestinal tract currently provides some of the strongest clinical observations. Fecal MNP concentrations are markedly elevated in patients with inflammatory bowel disease and appear to increase with disease severity [13,14]. In a cohort study of 188 resected intestinal tumors, in situ Raman and hyperspectral imaging detected MPs in 56.4% of specimens, with particle-positive tumors exhibiting greater local inflammation and more pronounced histopathological alterations [15]. Similar associations have been reported beyond the intestine. In patients undergoing carotid endarterectomy, polyethylene and polyvinyl chloride particles identified within atherosclerotic plaques were associated with increased plaque inflammation and a substantially higher risk of major cardiovascular events during follow-up [16]. Studies of the central nervous system have also reported elevated polymer burdens in the frontal cortex, particularly among individuals with neurodegenerative disorders [17]. Moreover, MNP concentrations were found to be higher in brain tumor tissues than in healthy brain samples and were associated with local pathological characteristics [18]. Of course, these findings should be interpreted cautiously. Most available human studies are cross-sectional, retrospective, or based on postmortem specimens and therefore cannot establish causality. Reverse causation, disease-related changes in particle retention, and analytical contamination remain important confounding factors. Nevertheless, the repeated detection of MNPs in diseased tissues across multiple organ systems raises an important mechanistic question: Through which biological pathways might chronic MNP exposure influence disease susceptibility and progression?

Current evidence suggests that the gastrointestinal tract is a key biological interface linking environmental exposure to systemic toxicity [19]. As the primary route of dietary particle uptake, it receives the highest continuous burden of MNPs, where particles encounter a dynamic luminal environment rich in enzymes, bile salts, microbial metabolites, and mucus. These interactions drive rapid biomolecular corona formation and remodeling, which in turn modulates particle transport, cellular uptake, and bioactivity [20,21]. The intestinal mucosa represents the largest epithelial and immune interface with the external environment and hosts a dense microbial ecosystem. Consequently, perturbations in barrier integrity, immune homeostasis, or microbiota composition may extend beyond the gut via integrated vascular, immune, endocrine, metabolic, and neural pathways. Emerging evidence further indicates that MNP exposure can disrupt microbial metabolism, impair epithelial barrier function, and induce immune and metabolic dysregulation, thereby triggering systemic effects through gut–organ axes rather than particle translocation alone [21,22]. The mechanistic understanding of MNP toxicity has evolved substantially over the past decade. Early studies primarily focused on physical abrasion, oxidative stress, and inflammatory injury. More recent work has revealed a much broader spectrum of biological effects, including protein corona formation, microbiome reprogramming, metabolic disruption, immune dysregulation, mitochondrial dysfunction, ferroptosis, and altered intercellular communication [23,24]. At the same time, several important questions remain unresolved. It remains unclear whether NPs and larger MPs follow fundamentally different toxicological pathways because of their distinct uptake mechanisms and tissue distribution patterns. The role of dynamic biomolecular coronas in determining particle fate and biological identity remains incompletely understood. The safety of biodegradable polymers, including polylactic acid (PLA), also remains under debate, as emerging evidence suggests that degradation products may induce metabolic disturbances under certain conditions [25]. Furthermore, the mechanisms by which localized intestinal responses are translated into systemic pathological outcomes across distant organs remain poorly defined. Rather than acting as independent causes of disease, current evidence suggests that MNPs are more likely to be the key factor in the induction and promotion of the disease. By disrupting mucosal barriers, altering microbial ecosystems, and influencing host signaling pathways, these particles may increase susceptibility to genetic, dietary, and environmental stressors that contribute to chronic disease development. Therefore, this review proposes a fundamental paradigm shift: MNP toxicity is not primarily driven by the inert physical accumulation of particles in distant tissues, but by their ability to act as chronic disruptors of intestinal ecology. The gut serves as a “biological amplifier”, translating localized, low-dose particulate exposure into systemic metabolic and immune dysfunction. Understanding this shift is critical, as it dictates that the true health risks of MNPs cannot be captured by measuring tissue burdens alone, but must be evaluated through the lens of gut-mediated systemic dysregulation. In this review, we synthesize recent advances in experimental and mechanistic studies, with particular emphasis on zebrafish and murine models, to examine how MNPs interact with the intestinal environment and how these interactions propagate across systemic organ networks. We discuss emerging mechanisms ranging from protein corona evolution and microbiome remodeling to metabolic reprogramming and inter-organ communication. By integrating evidence from particle biology, microbiology, toxicology, and systems physiology, we aim to provide a gut-centered framework for understanding the progression from environmental exposure to systemic dysfunction and to identify key priorities for future risk assessment and intervention strategies.

## Physicochemical Determinants of MNP Toxicity: Integrating Size, Morphology, and Polymer Chemistry

MNP toxicity cannot be explained by particle size alone. Increasing evidence indicates that the biological behavior of MNPs is determined by the combined effects of particle size, morphology, and polymer chemistry. These physicochemical properties influence every stage of particle–host interaction, including biological transport, cellular uptake, tissue retention, and activation of stress responses [26,27]. Rather than acting independently, they continuously interact in vivo to determine the biological identity and toxicological profile of MNPs. Particle size primarily determines biological accessibility. NPs can cross epithelial barriers and interact directly with intracellular organelles, whereas larger MPs remain largely confined to the intestinal lumen and mucus layer, where they mainly exert mechanical and ecological effects [27]. Particle morphology further modifies these responses by affecting particle retention, epithelial contact, and immune recognition. At the same time, polymer composition and degradation behavior regulate surface reactivity, biomolecular interactions, and the release of degradation products or adsorbed contaminants. Together, these 3 dimensions provide a useful framework for understanding the diverse mechanisms underlying MNP toxicity.

### Size-dependent intracellular toxicity of NPs

Compared with MPs, NPs more readily penetrate the intestinal epithelium through clathrin-mediated endocytosis, caveolin-mediated endocytosis, and micropinocytosis [28]. Their biological behavior is not determined solely by intrinsic polymer properties but is strongly shaped by the rapid formation of a biomolecular corona after exposure to gastrointestinal fluids (Fig. 1A). This corona is highly dynamic and evolves as particles transit through different compartments of the digestive tract, continuously modifying receptor recognition, cellular uptake routes, and intracellular trafficking patterns [29,30].

**Fig. 1.**
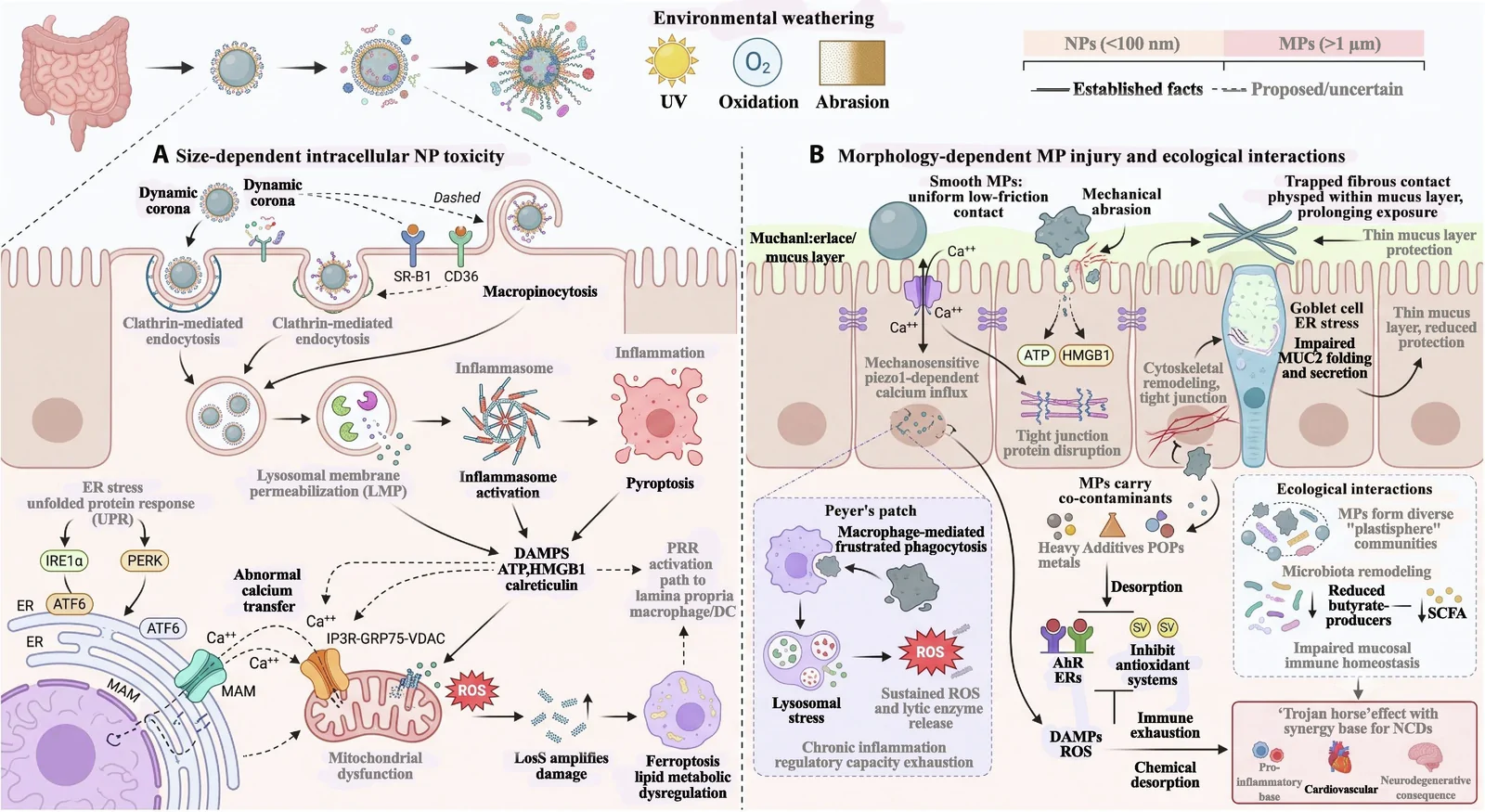
Mechanisms of size- and morphology-dependent gut toxicity for micro- and nanoplastics (MNPs). (A) Schematic diagram detailing size-dependent intracellular nanoplastic (NP) toxicity. Various endocytic pathways are shown leading to lysosomal membrane permeabilization, inflammasome activation, and subsequent pyroptosis. Also illustrated are endoplasmic reticulum (ER) stress pathways, abnormal calcium transfer causing mitochondrial dysfunction, and resulting ferroptosis, all contributing to a pro-inflammatory state and pattern recognition receptor (PRR) activation in the lamina propria. (B) Overview of morphology-dependent MP injury and ecological interactions. Panels illustrate mechanical effects from smooth and fibrous MPs on the mucus layer and Goblet cells, mechanosensitive Piezo1-dependent calcium influx, and tight junction disruption. It further depicts how MPs carry and release co-contaminants [heavy metals, additives, persistent organic pollutants (POPs)] affecting antioxidant systems and immune cells in Peyer’s patches, as well as microbiota remodeling and “plastisphere” formation, collectively driving a pro-inflammatory baseline and potential noncommunicable disease (NCD) risks via a “Trojan horse” effect. Dashed lines denote hypothetical, proposed, or indirect pathways that require further experimental validation. UPR, unfolded protein response; LMP, lysosomal membrane permeabilization; ROS, reactive oxygen species.

The composition and structure of this corona are further influenced by the environmental history of the particles prior to ingestion. Weathering processes such as ultraviolet irradiation, oxidation, and mechanical abrasion introduce oxygen-containing functional groups, increase surface roughness, and alter surface charge characteristics [31,32]. These physicochemical changes can affect the adsorption affinity for proteins, lipids, and bile components once inside the gastrointestinal lumen, thereby reshaping corona composition and its biological identity [24]. Experimental evidence suggests that corona-associated lipoproteins and lipid fractions may facilitate interactions with epithelial receptors, including scavenger receptor class B member 1 (SR-B1) and cluster of differentiation 36 (CD36) [33]. However, the extent to which these pathways contribute to NP uptake under physiological exposure conditions remains incompletely defined and may depend on particle aging status and local luminal conditions. Following ingestion, NPs undergo efficient cellular internalization and are frequently trafficked into endolysosomal compartments (Fig. 1A). Because of their limited degradability, progressive accumulation within lysosomes can lead to functional stress and membrane destabilization. Recent biophysical evidence suggests that NP–lysosome interactions may involve disruption of lipid bilayer integrity, contributing to lysosomal membrane permeabilization and leakage of lysosomal enzymes into the cytosol [34,35]. Release of proteases such as cathepsin B has been implicated in triggering downstream inflammatory signaling, including inflammasome activation [36,37]. Concurrently, NP exposure is associated with broader intracellular stress responses involving the endoplasmic reticulum (ER) and mitochondria [38–40]. ER stress may activate the unfolded protein response (UPR) pathways, including IRE1α, PERK, and ATF6 signaling branches. Sustained ER stress can promote abnormal calcium transfer between the ER and mitochondria through mitochondria-associated membrane (MAM) contact sites, involving channels such as IP3R–GRP75–VDAC complexes (Fig. 1A). This calcium imbalance contributes to mitochondrial dysfunction, including loss of membrane potential, impaired electron transport chain activity, and increased reactive oxygen species (ROS) production [38,41,42]. In parallel, oxidative stress may further exacerbate cellular damage and amplify inflammatory signaling.

Depending on exposure context and particle characteristics, these converging stress pathways may activate regulated cell death programs, including pyroptosis and ferroptosis [23,43]. Lysosomal dysfunction and cathepsin B release may promote inflammasome assembly, while mitochondrial ROS accumulation and lipid metabolic dysregulation may contribute to ferroptotic signaling [43,44]. Although these mechanisms have been repeatedly observed in experimental systems, their relative contribution and hierarchy likely vary across models and exposure conditions. Importantly, NP-induced epithelial injury is not strictly confined to intracellular compartments (Fig. 1). Cell death processes such as pyroptosis and ferroptosis may be accompanied by the release of damage-associated molecular patterns (DAMPs), including adenosine triphosphate (ATP), high mobility group box 1 (HMGB1), and calreticulin exposure on dying cell surfaces [45–47]. These signals can activate resident macrophages and dendritic cells in the lamina propria via pattern recognition receptors (PRRs) (Fig. 1A), thereby amplifying local inflammatory responses and potentially contributing to systemic immune signaling [33–35]. Whether this DAMP-driven amplification plays a dominant role under chronic low-dose human exposure remains uncertain and requires further investigation.

### Morphology-dependent mechanical injury and microbiota remodeling by MPs

Unlike NPs, MPs are generally too large to efficiently penetrate epithelial cells and therefore remain confined to the intestinal lumen or mucus layer (Fig. 1B). As a result, their toxicological profile is primarily driven by prolonged physical interaction with the mucosal surface rather than intracellular accumulation [21,48]. Continuous exposure of the intestinal epithelium to MPs may generate persistent mechanical stress, including repeated friction against microvilli. This process has been shown to activate mechanosensitive pathways such as Piezo1-dependent calcium influx in enterocytes, which can contribute to cytoskeletal remodeling and disruption of tight junction proteins, thereby weakening epithelial barrier integrity [49].

Beyond particle size, morphology further determines the extent and type of tissue injury. Smooth spherical MPs typically generate relatively uniform and low-friction contact with epithelial surfaces (Fig. 1B). In contrast, irregular fragments can produce localized mechanical abrasion of the epithelial membrane, which may trigger the release of intracellular DAMPs, including ATP and HMGB1, thereby amplifying local inflammatory signaling beyond purely mechanical damage (Fig. 1B). Fibrous MPs, due to their high aspect ratio, are more likely to become physically trapped within the mucus layer, prolonging epithelial exposure and reducing clearance [50,51]. This persistent contact can exacerbate ER stress in goblet cells, impairing MUC2 folding and secretion, and ultimately contributing to mucus layer thinning and reduced barrier protection [30,40]. In parallel, macrophage- and M cell-mediated sampling of MPs in Peyer’s patches provides an additional route for immune interaction [29,52]. However, because MPs are nondegradable and often exceed optimal phagocytic size, they can induce “frustrated phagocytosis” in macrophages. This incomplete engulfment is associated with lysosomal stress and sustained release of ROS and lytic enzymes, which may contribute to chronic, low-grade inflammatory activation and gradual exhaustion of local immune regulatory capacity [53–55]. Importantly, persistent mucus layer disruption further amplifies these effects by perturbing the goblet cell–mucin axis. Mechanical irritation and cellular stress within goblet cells impair MUC2 synthesis and secretion, weakening mucus renewal and exposing the epithelium to prolonged particle and microbial contact [55–57]. This disruption creates a feedback loop in which barrier damage increases particle retention, further intensifying mechanical and immune stress.

Beyond direct mechanical and immune effects, MPs also alter the intestinal microenvironment through ecological interactions. Their hydrophobic surfaces facilitate microbial adhesion and biofilm formation, leading to the development of plastisphere communities that differ from the surrounding gut microbiota [58–60]. These biofilms can shift microbial composition and metabolic activity, often characterized by a reduction in butyrate-producing bacteria and decreased short-chain fatty acid (SCFA) availability (Fig. 1B). Given the role of butyrate in supporting epithelial energy metabolism and regulatory T cell differentiation, such changes may contribute to impaired mucosal immune homeostasis [10,61]. In addition, MPs act as carriers for environmental co-contaminants, including heavy metals, plastic additives, and persistent organic pollutants (POPs), which may be adsorbed during environmental exposure and subsequently released under gastrointestinal conditions [62–64]. This localized desorption can increase chemical exposure at the epithelial interface. Mechanistically, released contaminants may exert additional toxicity through inhibition of antioxidant defense systems via interaction with sulfhydryl groups, as well as activation of xenobiotic-responsive nuclear receptors such as the aryl hydrocarbon receptor (AhR) and estrogen receptors (ERs) [65,66]. In this context, mechanical injury and chemical exposure act in a coupled manner: Epithelial barrier disruption may facilitate deeper penetration of reactive chemical species, thereby amplifying oxidative stress and molecular damage beyond what is induced by physical abrasion alone. Crucially, this “Trojan horse” effect suggests that MNPs do not act alone but synergize with ubiquitous environmental pollutants to drive chronic noncommunicable diseases (NCDs). The co-exposure of MNPs with heavy metals or PM2.5 can continuously deplete systemic antioxidant reserves, creating a pro-inflammatory baseline that accelerates the pathogenesis of cardiovascular and neurodegenerative NCDs (Fig. 1B).

### Polymer chemistry and degradation-dependent metabolic perturbation

Besides particle size and morphology, polymer chemistry is another major determinant of MNP toxicity. Polymer composition influences surface hydrophobicity, crystallinity, degradation kinetics, and interactions with biological molecules, thereby shaping both particle persistence and host responses [67,68]. These properties are particularly relevant for biodegradable plastics such as PLA. Although PLA exhibits lower environmental persistence than conventional polyolefins, accumulating evidence suggests that biodegradability should not be considered synonymous with biological safety. Instead, the degradation process itself appears to be an important determinant of toxicity (Fig. 2).

**Fig. 2.**
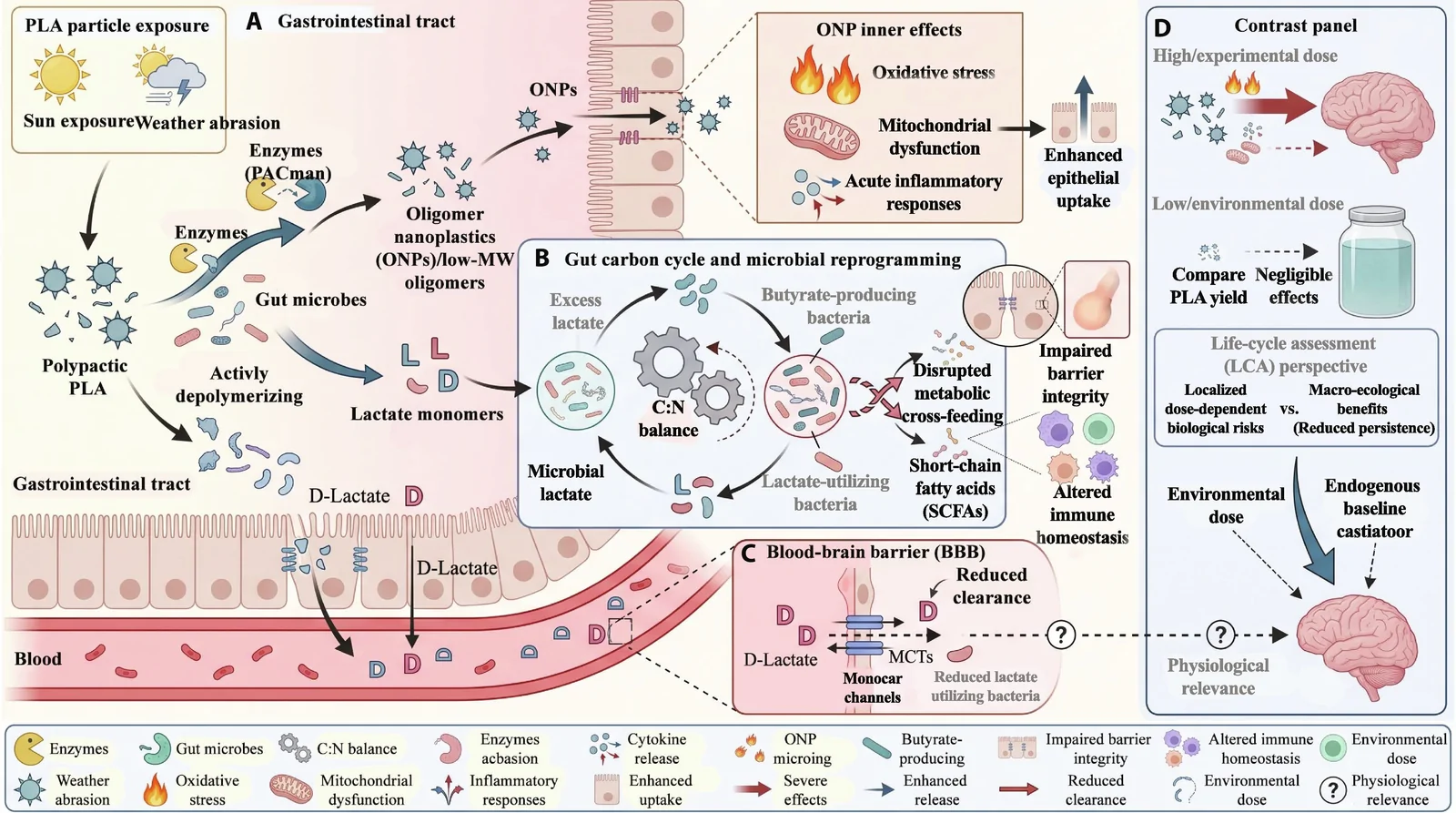
Gastrointestinal degradation and systemic biological impacts of polylactic acid (PLA) particles. (A) Schematic diagram of PLA depolymerization into oligomer nanoplastics (ONPs) and lactate monomers, inducing epithelial oxidative stress and acute inflammatory responses. (B) Overview of gut carbon cycle disruption and microbial reprogramming driven by excess lactate, altering short-chain fatty acid (SCFA) production and barrier integrity. (C) Diagram of d-lactate translocation across the blood–brain barrier (BBB) via monocarboxylate transporters (MCTs). (D) Contrast panel comparing high experimental doses with low environmental exposures, alongside a life-cycle assessment (LCA) perspective on localized biological risks versus macro-ecological benefits. Dashed lines denote hypothetical, proposed, or indirect pathways that require further experimental validation. MW, molecular weight.

Unlike conventional plastics, which remain relatively stable in the gastrointestinal tract, PLA undergoes enzymatic and microbial depolymerization after ingestion. Recent studies further indicate that this process is often incomplete, generating not only lactate monomers but also low-molecular-weight oligomers and oligomer nanoplastics (ONPs) (Fig. 2A). These degradation intermediates exhibit greater surface reactivity and cellular accessibility than the parent particles and have been associated with enhanced epithelial uptake, oxidative stress, mitochondrial dysfunction, and acute inflammatory responses [69,70]. Environmental weathering, particularly photoaging, may further accelerate polymer fragmentation before ingestion, thereby increasing the release of ONPs and other reactive degradation products during gastrointestinal digestion [71,72]. Beyond particle fragmentation, PLA degradation also reshapes gut microbial metabolism. Lactate released during depolymerization enters the intestinal carbon cycle and serves as an additional carbon source for resident microorganisms. Rather than simply altering microbial composition, this excess carbon input appears to reprogram microbial metabolism by disturbing the carbon-to-nitrogen balance and disrupting metabolic cross-feeding within the gut ecosystem [70,73,74]. Experimental studies have reported reduced abundance of lactate-utilizing and butyrate-producing bacteria, accompanied by decreased SCFA production, impaired epithelial barrier integrity, and altered immune homeostasis (Fig. 2B). Because mammalian cells metabolize l- and d-lactate differently, excessive accumulation of d-lactate under certain conditions has also been proposed to contribute to local metabolic disturbances [75–77]. Besides, d-lactate easily escapes the gut and crosses the blood–brain barrier (BBB) via monocarboxylate transporters (MCTs) (Fig. 2C). Reduced activity of lactate-utilizing bacteria may further limit lactate clearance [70,78,79], although the physiological relevance of this mechanism under environmentally realistic exposure conditions remains to be established. Crucially, these metabolic perturbations must be interpreted with quantitative caution. The endogenous lactate pool continuously generated by human gut microbial fermentation is substantial, typically reaching millimolar concentrations in the colon. At environmentally realistic exposure levels—supported by human biomonitoring data and exposure assessments from Qiu and Huang’s related research indicating that internal systemic tissue burdens and daily dietary intakes remain strictly in the low dose range (0.01 to 0.2 mg/kg body weight/day) [69,80]—the theoretical yield of lactate derived from intestinal PLA degradation is negligible compared to this endogenous baseline (Fig. 2D). Under such low-dose conditions, biological systems may exhibit a stimulus response—whereby trace amounts of degradation intermediates trigger mild, adaptive metabolic adjustments or transient microbial preconditioning rather than overt toxicity. In contrast, the severe carbon–nitrogen imbalances and d-lactate accumulation reported in recent in vivo studies are predominantly driven by acute experimental doses (e.g., 25 mg/kg body weight/day) that exceed real-world environmental human intake by several orders of magnitude [70,81].

Importantly, the toxicity of biodegradable plastics is not limited to PLA. Emerging evidence shows that poly (butylene adipate-co-terephthalate) (PBAT) and polyhydroxyalkanoates (PHAs) can also induce biological disturbances through polymer- and degradation-dependent mechanisms. During gastrointestinal digestion, PBAT oligomers can increase rather than degrade, and their migrants exhibit cytotoxicity toward Caco-2 cells while disrupting gut microbiota and SCFA metabolism, particularly acetate and butyrate production [82]. Similarly, PHA MNPs perturb the microbiome–metabolome–organ axis following both inhalation and ingestion, inducing respiratory and gut dysbiosis, systemic metabolic alterations, and hepatic transcriptional responses, although generally with lower toxicity than polypropylene (PP) MNPs [83]. A recent systematic review further indicates that biodegradable MNPs can alter microbial communities, inhibit growth and reproduction, and modify pollutant bioavailability, with toxicity strongly dependent on polymer type, particle characteristics, dose, and aging [84]. Collectively, these findings challenge the assumption that biodegradability equates to biological safety and highlight polymer-specific degradation products, chemical migrants, microbiome dysbiosis, metabolic reprogramming, and cross-organ signaling as emerging mechanisms of biodegradable MNP toxicity. Thus, navigating the safety of biodegradable plastics requires a balanced life-cycle assessment (LCA) perspective (Fig. 2D). While the transient bio-reactivity of intermediate degradation products (such as ONPs or specific monomers) under gastrointestinal conditions merits attention, these localized and dose-dependent biological risks must be carefully weighed against the profound macro-ecological benefits of these materials [70]. By obviously reducing environmental persistence and mitigating the irreversible, centuries-long accumulation of conventional MPs, biodegradable polymers remain a critical environmental solution. Future material design should focus on optimizing polymer formulations to retain robust environmental degradability while minimizing the biological reactivity of their transitional products under realistic human exposure scenarios.

## Gut-Centered Systemic Effects of MNPs: Multi-Organ Crosstalk

Although the gastrointestinal tract is the primary site of MNP exposure, increasing evidence suggests that intestinal toxicity is rarely confined to the gut [60,85]. Instead, disruption of the intestinal barrier initiates a series of interconnected biological events that extend to distant organs through immune, metabolic, microbial, and neural signaling pathways (Figs. 3 and 4). Rather than acting independently, these pathways interact extensively, allowing local epithelial injury to propagate into systemic inflammation and metabolic dysfunction. Current evidence therefore supports the concept that the gut functions as the central coordinator of MNP-induced systemic toxicity.

**Fig. 3.**
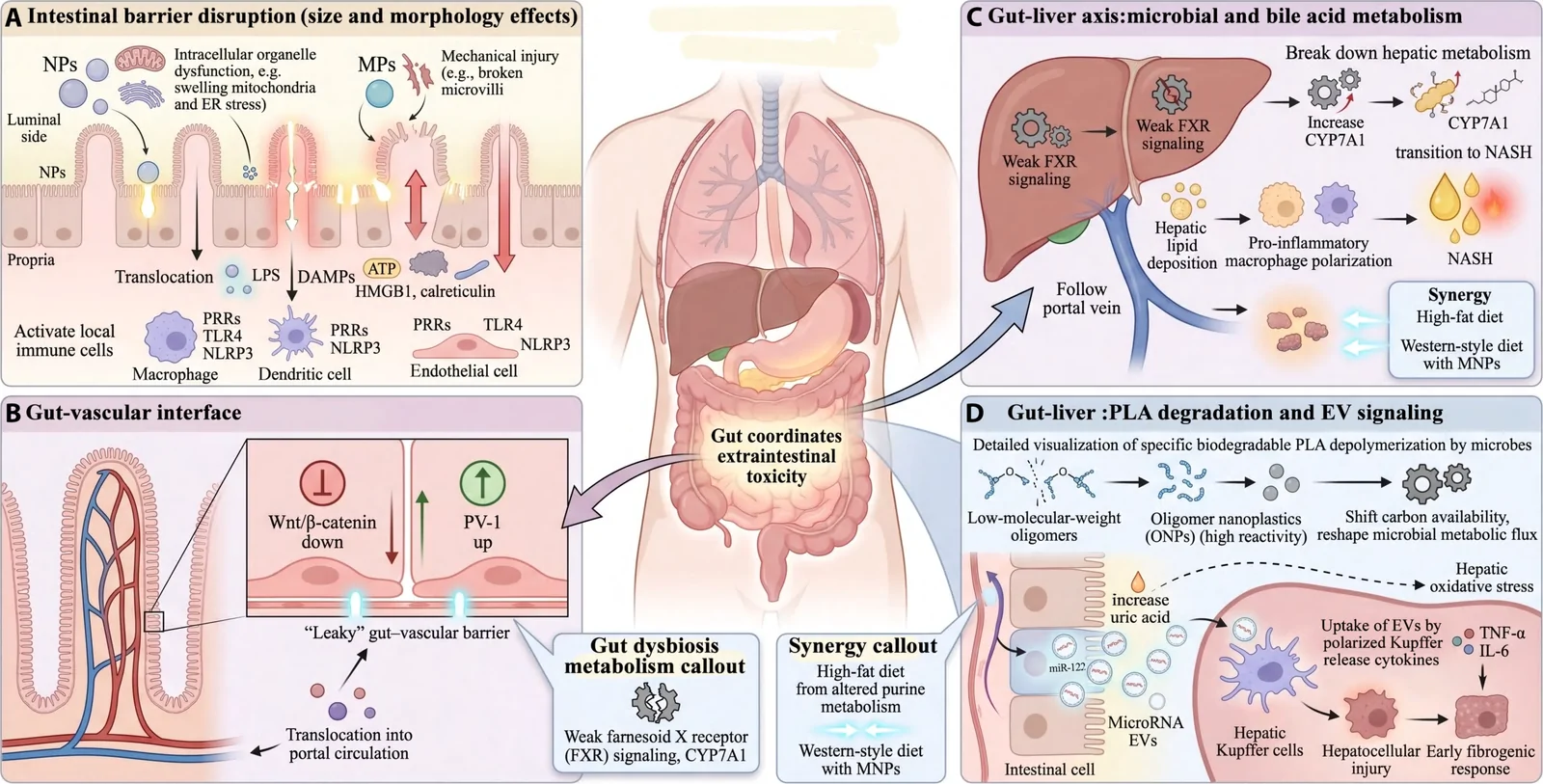
Gut-centered systemic effects of micro/nanoplastics (MNPs) via the gut–liver axis. (A) Schematic diagram of intestinal barrier disruption driven by MNP size and morphology, leading to local immune cell activation. (B) Overview of the gut–vascular interface illustrating increased permeability and MNP translocation into the portal circulation. (C) Schematic diagram of disrupted microbial and bile acid metabolism, exacerbating hepatic lipid deposition and the transition to nonalcoholic steatohepatitis (NASH). (D) Diagram detailing polylactic acid (PLA) degradation and extracellular vesicle (EV) signaling that induce hepatic fibrogenic responses. Dashed lines denote hypothetical, proposed, or indirect pathways that require further experimental validation. NP, nanoplastic; MP, microplastic; LPS, lipopolysaccharide; DAMP, damage-associated molecular pattern; PRR, pattern recognition receptor; TLR4, Toll-like receptor 4; NLRP3, NLR family pyrin domain containing 3; PV-1, plasmalemma vesicle-associated protein-1; FXR, farnesoid X receptor; CYP7A1, cytochrome P450 family 7 subfamily A member 1; ONP, oligomer nanoplastic; TNF-α, tumor necrosis factor-α; IL-6, interleukin-6.

**Fig. 4.**
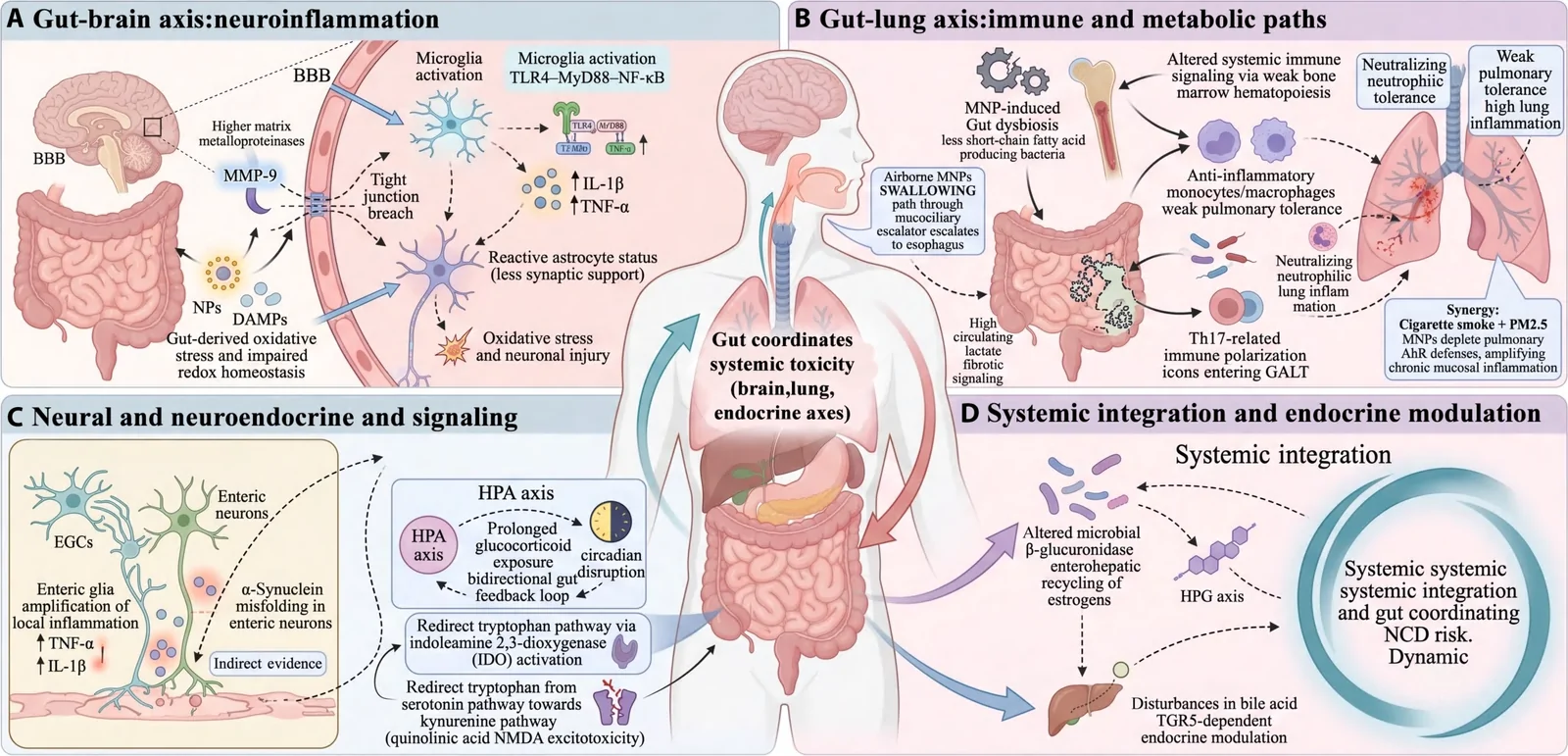
Gut-centered systemic effects of micro/nanoplastics (MNPs) via multi-organ crosstalk. (A) MNP-mediated neuroinflammation along the gut–brain axis. Schematic diagram detailing blood–brain barrier (BBB) compromise and microglial activation. (B) Schematic diagram of the gut–lung axis highlighting altered immune and metabolic pathways driven by gut dysbiosis. (C) Overview of neural and neuroendocrine signaling disruptions, including enteric nervous system activation and hypothalamic–pituitary–adrenal (HPA) axis interference. (D) Diagram of systemic integration and endocrine modulation coordinating noncommunicable disease (NCD) risks. Dashed lines denote hypothetical, proposed, or indirect pathways that require further experimental validation. DAMP, damage-associated molecular pattern; TLR4, Toll-like receptor 4; MyD88, myeloid differentiation primary response 88; NF-κB, nuclear factor κB; IL-1β, interleukin-1β; TNF-α, tumor necrosis factor-α; GALT, gut-associated lymphoid tissue; IDO, indoleamine 2,3-dioxygenase; HPG, hypothalamic–pituitary–gonadal.

### Intestinal barrier disruption as the initiating event of systemic toxicity

The intestinal epithelium forms the first biological barrier separating luminal contents from the internal environment. As discussed in the Physicochemical Determinants of MNP Toxicity: Integrating Size, Morphology, and Polymer Chemistry section, MNP exposure disrupts epithelial integrity through distinct mechanisms that depend on particle size, morphology, and polymer chemistry. NPs primarily induce intracellular organelle dysfunction, whereas MPs mainly cause chronic mechanical injury and microbial dysbiosis. Despite these mechanistic differences, both ultimately impair epithelial barrier function and increase intestinal permeability (Fig. 3A).

Loss of barrier integrity permits the translocation of bacterial products, microbial metabolites, inflammatory cytokines, and, under some conditions, small plastic particles into the lamina propria and systemic circulation. Increased circulating lipopolysaccharide (LPS), ATP, HMGB1, and other DAMPs activate resident macrophages, dendritic cells, and endothelial cells through PRRs, including Toll-like receptors and the *NLRP3* inflammasome (Fig. 3A). These local immune responses may subsequently develop into low-grade systemic inflammation [46,68,86,87]. Although most evidence derives from rodent models, barrier dysfunction is widely considered the common upstream event linking intestinal MNP exposure with extraintestinal pathology.

### Gut-derived metabolites remodel hepatic metabolism

Among extraintestinal targets, the liver is generally considered the first major organ exposed to gut-derived MNPs because of its direct anatomical connection to the intestine via the portal vein (Fig. 3). However, hepatic responses are not driven by particle deposition alone [88]. Current evidence points to a stepwise failure of intestinal barrier and metabolic control systems that together determine the extent of liver exposure.

One key step occurs at the level of the gut–vascular interface. Experimental work suggests that NPs can interfere with Wnt/β-catenin signaling in intestinal endothelial cells, which is closely linked to vascular integrity and repair. Down-regulation of this pathway has been associated with increased expression of plasmalemma vesicle-associated protein (PV-1), a marker of endothelial fenestration, which together may promote a more permeable vascular phenotype often described as a “leaky” gut–vascular barrier [89,90]. Under these conditions, gut-derived microbial products such as LPS, along with smaller plastic particles, may gain easier access to the portal circulation (Fig. 3B), contributing to hepatic exposure to inflammatory and xenobiotic stress signals [46,91,92]. At the same time, MNP exposure has been linked to shifts in gut microbial communities that reduce bacteria expressing bile salt hydrolase (BSH). This disruption limits bile acid deconjugation and may weaken farnesoid X receptor (FXR) signaling, which normally helps maintain bile acid and lipid homeostasis. Reduced FXR activity is associated with increased *CYP7A1*-driven bile acid synthesis and intracellular bile acid accumulation while also promoting lipogenic pathways involving SREBP-1c and fatty acid synthase (FAS) (Fig. 3C), thereby favoring hepatic lipid deposition and increasing vulnerability to metabolic stress [10,93–95]. In real-world scenarios, this MNP-induced lipid dysregulation frequently interacts with unhealthy dietary patterns. Co-exposure to MNPs and a high-fat or Western-style diet can synergistically overwhelm hepatic lipid clearance and amplify pro-inflammatory macrophage polarization, thereby accelerating the transition from simple steatosis to severe metabolic NCDs, such as non-alcoholic steatohepatitis (NASH) [96].

Beyond these general microbial and bile acid-related effects, biodegradable polymers such as PLA may introduce additional layers of metabolic disturbance through their degradation products (Fig. 3D). Gut microbial depolymerization of PLA can shift intestinal carbon availability and reshape microbial metabolic flux. In parallel, changes in purine metabolism and increased circulating uric acid have been reported in experimental models, accompanied by activation of oxidative stress pathways in the liver [69,70,81,97]. Uric acid accumulation has been associated with *NLRP3* inflammasome activation, redox imbalance, and lipid metabolic dysregulation in animal studies [98], although the strength of these effects under environmentally relevant exposure conditions remains uncertain. Gut–liver communication is also mediated by extracellular vesicle (EV) signaling (Fig. 3D). Under MNP-induced stress, intestinal epithelial cells may release EVs with altered microRNA profiles, including candidates such as *miR-155* and *miR-122*. After uptake by hepatic Kupffer cells, these signals have been proposed to modulate suppressor of cytokine signaling (SOCS) pathways and shift macrophage polarization toward a more pro-inflammatory phenotype. Activated Kupffer cells can then release cytokines such as *TNF-α* and *IL-6*, which amplify inflammatory signaling in hepatocytes and may contribute to downstream *STAT3* activation, hepatocellular injury, and early fibrogenic responses [39,60,99,100].

Overall, current findings suggest that MNP-related liver injury is more consistently explained by disrupted gut barrier function, altered microbial and bile acid metabolism, and immune–metabolic signaling along the gut–liver axis, rather than by direct accumulation of plastic particles in hepatic tissue [92,101]. However, most mechanistic evidence still comes from controlled experimental systems with relatively high exposure levels, and its relevance to chronic low-dose human exposure requires further validation.

### Gut microbial and neural signaling contributes to brain dysfunction

Communication between the gut and the central nervous system provides a key route through which MNPs may influence brain function (Fig. 4A). Current evidence supports a multi-layered model integrating barrier dysfunction, immune activation, and microbiota-derived metabolic signaling, with additional modulation by neuroendocrine and neural pathways.

At the intestinal barrier level, MNP exposure has been associated with impaired redox homeostasis, including attenuation of *Nrf2*-dependent antioxidant responses. This shift increases susceptibility to oxidative stress and promotes the release of ROS and matrix metalloproteinases, particularly MMP-9. These factors are linked to degradation of tight junction proteins such as claudin-5 and ZO-1 in the BBB, contributing to increased permeability rather than overt structural collapse [46,88,102,103]. Evidence for direct particle translocation into brain tissue remains limited and inconsistent across experimental systems. Barrier dysfunction facilitates the entry of peripheral inflammatory mediators into the central nervous system, where they interact with resident immune cells. Circulating cytokines and DAMPs may activate microglia via *TLR4–MyD88–NF-κB* signaling [104–106]. Across animal models, this is commonly accompanied by increased *IL-1β* and *TNF-α* expression, oxidative stress, and neuronal injury markers (Fig. 4A). Microglial activation is closely coupled to astrocytic responses, in which microglia-derived *IL-1α*, *TNF-α*, and *C1q* have been implicated in inducing reactive astrocyte states with reduced synaptic support capacity [107]. However, the stability and molecular definition of these astrocytic phenotypes in MNP-related exposure remain under refinement. In parallel, gut microbiota–derived metabolic disruption provides an additional mechanistic axis. MNP-associated dysbiosis is frequently linked to reduced SCFA production, particularly butyrate, which may impair microglial immune tolerance [9,108]. Inflammatory signaling also induces indoleamine 2,3-dioxygenase (IDO), redirecting tryptophan metabolism from serotonin synthesis toward the kynurenine pathway (Fig. 4C). This shift increases neuroactive metabolites such as quinolinic acid, which can promote *N*-methyl-D-aspartic acid (NMDA) receptor overactivation and excitotoxic stress. These immune–metabolic interactions likely operate in a coupled manner rather than as independent pathways. Neural signaling pathways further integrate intestinal stress into central regulation. Chronic intestinal irritation may activate vagal afferent signaling, transmitting peripheral inflammatory states to brain regions involved in autonomic control. Within the enteric nervous system, enteric glial cells (EGCs) may amplify local inflammatory signaling under sustained exposure, releasing cytokines such as *IL-1β* and *TNF-α* that destabilize neuronal homeostasis [72,80,109]. This environment has been proposed to facilitate α-synuclein misfolding in enteric neurons, with potential propagation along the vagus nerve [110,111]; however, evidence under environmentally relevant exposure conditions remains largely indirect.

Beyond neuroimmune and neural routes, MNP-induced gut dysbiosis may influence systemic endocrine regulation (Fig. 4D). Altered microbial β-glucuronidase activity can modify enterohepatic recycling of estrogens and endocrine disruptors, potentially affecting hypothalamic–pituitary–gonadal (HPG) axis signaling [112]. Disturbances in bile acid metabolism may further impair TGR5-dependent endocrine and metabolic regulation [113]. These pathways likely act as modulatory components within a broader neuroimmune framework. Finally, sustained low-grade intestinal inflammation may function as a chronic stress input to central neuroendocrine circuits. Vagal and humoral signaling to the hypothalamus may contribute to prolonged activation of the hypothalamic–pituitary–adrenal (HPA) axis, resulting in sustained glucocorticoid exposure [114]. This state is associated with hippocampal vulnerability, immune dysregulation, and further impairment of gut barrier integrity, forming a bidirectional gut–brain feedback loop [72,115]. Emerging evidence also suggests that disruption of circadian coordination, through altered cortisol rhythms and microbiome oscillations (Fig. 4C), may further destabilize host–microbe homeostasis and amplify inflammatory tone [116,117]. However, the quantitative contribution of these interconnected pathways under chronic environmental exposure remains to be defined.

### Gut dysbiosis influences pulmonary function and endocrine homeostasis

Beyond the liver and brain, accumulating evidence suggests that intestinal MNP exposure can also affect pulmonary function through the gut–lung axis (Fig. 4B). Following ingestion, MNP-induced dysbiosis and intestinal inflammation alter systemic immune signaling, creating conditions that favor pulmonary inflammatory responses even in the absence of substantial particle accumulation within the lung. Reduced abundance of SCFA-producing bacteria is considered a central event in this process [118,119]. Because gut-derived SCFAs regulate bone marrow hematopoiesis and promote the generation of anti-inflammatory monocytes and macrophages (Fig. 4B), MNP-associated depletion of these metabolites may impair pulmonary immune tolerance and increase susceptibility to inflammatory lung injury [118,120].

MNP exposure may also reshape adaptive immune responses along the gut–lung axis. Experimental studies have shown that intestinal dysbiosis induced by MNPs promotes the expansion of pro-inflammatory bacterial taxa and enhances *Th17*-related immune polarization within gut-associated lymphoid tissues (Fig. 4B). These activated immune cells and their cytokine products can subsequently enter systemic circulation and contribute to neutrophilic inflammation in pulmonary tissues, a mechanism that has been linked to aggravated airway inflammation and chronic respiratory disease phenotypes in animal models [121]. Additional metabolite-mediated pathways have also been proposed. MNP-induced alterations in microbial metabolism can reduce the production of indole derivatives, which normally activate AhR signaling and help maintain epithelial antioxidant defenses. Impaired AhR signaling may increase pulmonary vulnerability to oxidative stress [118,122]. Changes in microbial fermentation patterns have likewise been associated with elevated circulating lactate levels (Fig. 4B), which may contribute to profibrotic signaling through activation of the *HIF1α/PTBP1* pathway [123]. Although these mechanisms remain incompletely understood, they suggest that MNP-induced microbial dysregulation can influence lung function through multiple metabolic routes. Notably, interactions between the respiratory tract and the gastrointestinal tract may further amplify exposure. Airborne MNPs trapped within airway mucus are continuously cleared by the mucociliary escalator and subsequently swallowed, providing an additional source of gastrointestinal exposure [124,125]. This process links inhalation and ingestion pathways and may reinforce intestinal MNP burden, thereby strengthening downstream gut–lung signaling. Taken together, current evidence indicates that pulmonary effects associated with MNP exposure arise predominantly from gut-derived immune and metabolic disturbances rather than from direct particle deposition alone, although the relative contribution of these pathways under chronic environmental exposure remains to be established. This interplay is further complicated by concurrent exposure to cigarette smoke and atmospheric particulate pollution. Smoking not only impairs mucociliary clearance—increasing the retention time of inhaled MNPs—but also introduces toxic co-contaminants like polycyclic aromatic hydrocarbons. The synergistic interaction between MNPs, smoke-derived toxins, and PM2.5 severely depletes pulmonary AhR-dependent antioxidant defenses, amplifying chronic mucosal inflammation (Fig. 4B). This multi-hit environmental assault plays a critical role in the exacerbation of respiratory NCDs, including asthma and chronic obstructive pulmonary disease (COPD) [126,127].

Current evidence therefore supports a mechanistic framework in which the gut functions as the primary upstream regulator of systemic MNP toxicity [68]. Nevertheless, most mechanistic studies have been performed in rodents using relatively high exposure concentrations [7]. Future work should integrate environmentally relevant exposure models, human cohort studies, and multi-omics approaches to determine the relative contribution of individual gut-derived pathways and to identify the mechanisms most relevant to human health [128].

## Experimental Platforms for Resolving MNP Toxicity Mechanisms

Mechanistic understanding of MNP toxicity depends heavily on the experimental models used. Different platforms capture distinct aspects of particle behavior, ranging from early epithelial interactions and immune responses to microbiota-mediated signaling and long-term systemic outcomes. As a result, apparent inconsistencies across studies often reflect differences in model architecture, exposure design, and biological resolution rather than genuine contradictions in toxicity mechanisms [7]. A major challenge for the field is therefore not simply generating more data but selecting models that match the biological question being addressed (Fig. 5).

**Fig. 5.**
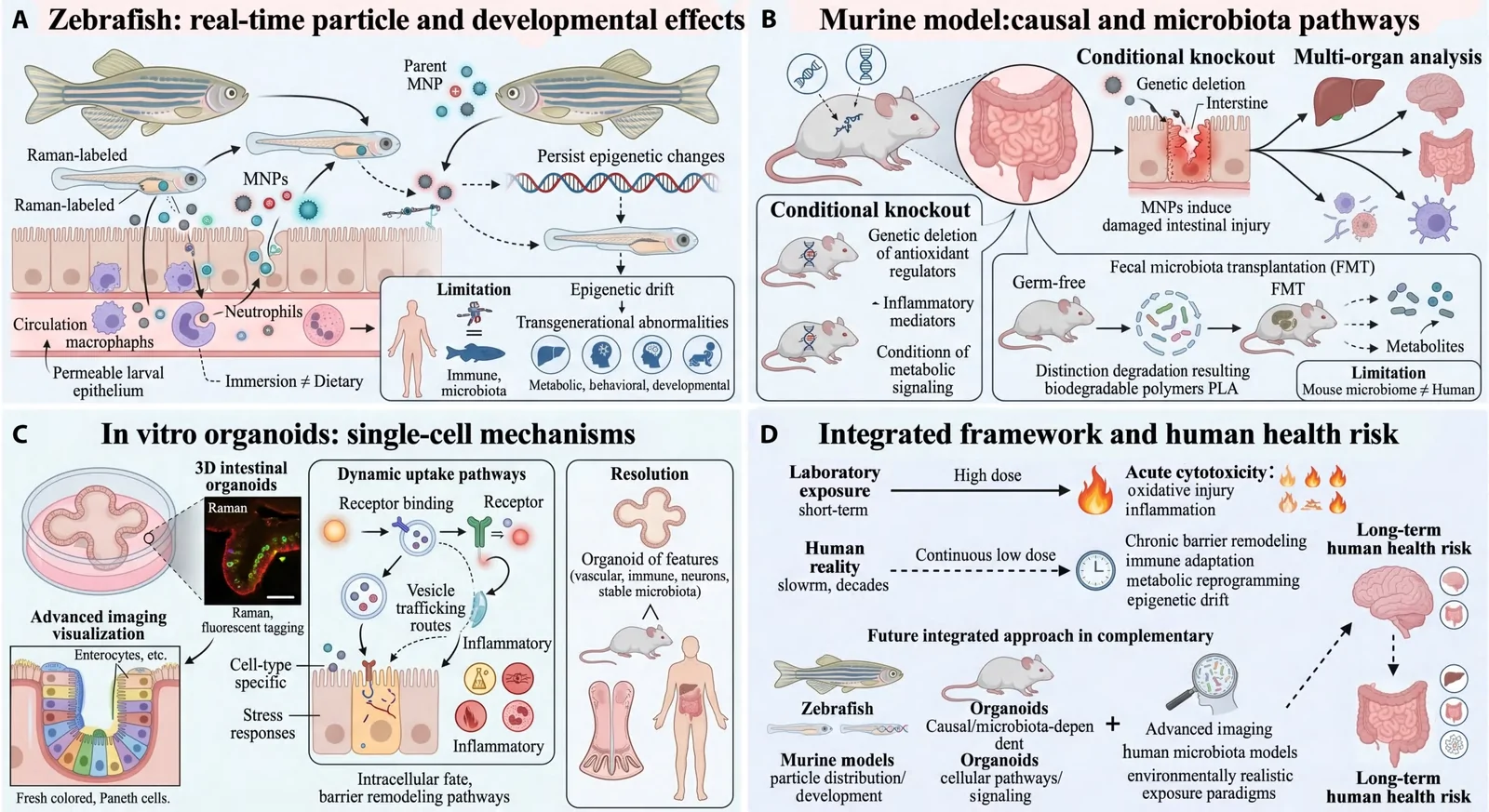
Experimental models and integrated frameworks for assessing micro/nanoplastic (MNP) toxicity. (A) Zebrafish models used for real-time particle tracking and evaluating developmental and epigenetic effects. Schematic diagram of in vivo MNP exposure and transgenerational abnormalities shown in the right panel. (B) Schematic diagram of murine models utilizing conditional knockouts and fecal microbiota transplantation (FMT) to investigate causal pathways and microbiota interactions. (C) Overview of in vitro 3-dimensional (3D) organoid platforms for elucidating single-cell mechanisms and dynamic cellular uptake pathways. (D) Diagram of an integrated framework combining complementary biological models to translate short-term laboratory findings into long-term human health risks. Dashed lines denote hypothetical, proposed, or indirect pathways that require further experimental validation.

### Zebrafish (*Danio rerio*) models for tracking particle transport and early biological responses

Zebrafish have become an important platform for investigating the early biological behavior of MNPs because their optical transparency permits direct visualization of particle distribution (Fig. 5A), epithelial translocation, and immune cell recruitment in living organisms [129,130]. Combined with fluorescent reporter lines, this system provides exceptional spatiotemporal resolution and allows researchers to monitor dynamic host–particle interactions that are difficult to capture in mammalian models [131,132].

These advantages are particularly valuable for studying physicochemical determinants of toxicity. Differences in particle size, surface charge, and morphology can be linked directly to tissue localization and inflammatory responses in vivo [133,134]. Zebrafish are also well suited for evaluating developmental and transgenerational effects because of their short reproductive cycle [135,136]. Several studies have reported that parental MNP exposure induces epigenetic alterations that persist in subsequent generations and are associated with metabolic, behavioral, or developmental abnormalities [137]. Despite these strengths, several limitations complicate interpretation (Fig. 5A). The gastrointestinal anatomy, immune system organization, and microbiota composition of zebrafish differ substantially from those of mammals [138]. Particle uptake is often enhanced by the relatively permeable epithelial structures present during early developmental stages, which may exaggerate translocation compared with adult mammalian intestines [136]. Exposure protocols also frequently rely on aquatic immersion rather than dietary ingestion, creating uncertainty regarding real-world relevance [139]. As a result, zebrafish are highly effective for identifying biological processes and generating mechanistic hypotheses, but they are less suitable for predicting human exposure outcomes directly.

### Murine models for establishing causal pathways

Murine models remain the principal system for investigating causal mechanisms of MNP toxicity because they allow genetic manipulation, controlled exposure, and multi-organ analysis within an intact mammalian physiology (Fig. 5B). Conditional knockout approaches have been particularly useful for identifying pathways that link intestinal injury to systemic disease [140]. For example, tissue-specific deletion of antioxidant regulators, inflammatory mediators, or metabolic signaling molecules has helped clarify how intestinal disturbances contribute to liver, brain, and immune dysfunction after MNP exposure [86]. Germ-free and fecal microbiota transplantation models provide an additional layer of mechanistic resolution by distinguishing microbiota-dependent effects from host-derived responses [141]. These approaches are especially important for biodegradable polymers such as PLA, where toxicity may depend on microbial degradation and metabolite production rather than on the particles themselves [97,142].

At the same time, murine studies face several challenges that affect translational interpretation (Fig. 5B). The composition of the mouse microbiome differs markedly from that of humans, particularly with respect to bile acid metabolism, carbohydrate utilization, and immune regulation [143]. Experimental exposure levels are often substantially higher than estimated human intake, raising concerns that acute toxicity endpoints may not accurately reflect chronic environmental exposure [144]. Housing conditions, diet, cage effects, and vendor-specific microbiota can further influence outcomes and contribute to poor reproducibility across laboratories. Humanized microbiota models partially address these issues but do not fully reproduce the complexity and stability of the human intestinal ecosystem [145]. Consequently, murine models remain indispensable for mechanistic validation, yet their findings should be interpreted as evidence of biological plausibility rather than direct estimates of human risk.

### Organoid and advanced in vitro systems for cellular-level mechanisms

Many of the key questions in MNP toxicology involve cellular events that are difficult to dissect in whole-animal models. These include epithelial uptake pathways, intracellular trafficking, barrier remodeling, and cell-type-specific stress responses [146]. Three-dimensional (3D) intestinal organoids provide a useful complement because they preserve major epithelial cell populations while allowing precise experimental control (Fig. 5C). When combined with advanced imaging approaches, including Raman-labeled or fluorescently tagged particles, organoid systems can resolve receptor-dependent uptake pathways, vesicular transport routes, and intracellular fate at single-cell resolution [147,148]. Because circulating immune cells, endocrine signals, and microbial variability can be minimized experimentally, organoids often provide greater mechanistic clarity than whole-animal studies.

However, organoids also introduce important constraints. Most systems lack vascular networks, resident immune cells, enteric neurons, and a stable microbiota [149,150]. As a result, they cannot reproduce the complex gut–liver, gut–brain, or gut–immune interactions that increasingly appear central to MNP toxicity (Fig. 5C). Particle exposure may also differ substantially from the physiological conditions present in the intestinal lumen, where mucus turnover, digestive enzymes, and microbial metabolism continuously modify particle properties [151]. Therefore, organoid findings should be interpreted as mechanistic evidence at the cellular level rather than as complete representations of organismal responses.

### Bridging the gap between experimental exposure and human reality

A central limitation of current MNP research is that mechanistic resolution and physiological relevance are rarely achieved within the same model. Organoids and cell-based systems are valuable for identifying uptake pathways, intracellular trafficking, and stress responses, but they cannot reproduce the complex interactions among microbiota, immune cells, circulation, and distant organs. Animal models provide this systemic context, yet the complexity of whole-organism responses often obscures the contribution of individual mechanisms [152,153]. Consequently, findings from different models are complementary rather than interchangeable, and conclusions drawn from a single experimental platform should be interpreted cautiously. This problem is further amplified by the discrepancy between environmental exposure and laboratory design. Human exposure is believed to occur continuously at relatively low levels over decades, whereas many experimental studies rely on short-term, high-dose exposures to accelerate phenotype development [7,154]. Importantly, human biomonitoring increasingly demonstrates that even chronic environmental exposure can result in measurable and substantial MNP burdens across the body. MNPs have been detected in human blood at a mean concentration of 1.6 μg/ml [155], indicating systemic bioavailability, while MNPs were detected in all examined human arterial samples at 118.66 ± 53.87 μg/g tissue, with even higher burdens in atherosclerotic coronary (156.50 ± 42.14 μg/g) and carotid arteries (133.37 ± 60.52 μg/g) [156]. More strikingly, postmortem analyses detected MNPs at median concentrations of 433 μg/g in liver, 404 μg/g in kidney, and 4,917 μg/g in brain tissue in 2024 samples, revealing substantial accumulation in internal organs [11]. Consistently, MNPs were detected in 99.4% of diseased brain samples and 100% of healthy brain samples, with higher burdens in peritumoral than healthy brain tissues [18]. MNP accumulation is also evident in diseased tissues, including colorectal tumors, where particle abundance reached 702.68 ± 504.26 particles/g, compared with 207.78 ± 154.12 particles/g in adjacent nontumor tissues [15], and hepatocellular carcinoma, where MPs were detected in 72% of tumor samples, with intratumoral polyvinyl chloride (PVC) burdens reaching 10 to 500 ng/g [157]. Such findings indicate that human MNP exposure is not simply a transient, low-level encounter but may involve persistent systemic transport and tissue-specific accumulation. Under these conditions, short-term high-dose experiments may preferentially reveal acute cytotoxic responses, including oxidative injury and inflammatory activation, while overlooking slower processes such as chronic barrier remodeling, immune adaptation, metabolic reprogramming, tissue-specific accumulation, and epigenetic drift (Fig. 5D).

Future progress will likely depend on integrated experimental frameworks that combine complementary model systems rather than relying on a single platform (Fig. 5D). Zebrafish can provide real-time information on particle biodistribution and developmental effects; murine models can establish microbiota-dependent and organ-specific causal relationships; organoid systems can define cellular uptake mechanisms and signaling pathways [158]. Integration of these approaches with human microbiome models, advanced imaging technologies (Fig. 5D), and environmentally realistic exposure paradigms may provide a more accurate framework for assessing long-term human health risks [9,72]. Taken together, the major challenge for future MNP research is no longer the identification of additional toxicity endpoints, but the development of experimental systems that better connect mechanistic observations with realistic human exposure scenarios. This transition will be essential for improving both risk assessment and the translational value of mechanistic toxicology studies.

## From Mechanistic Understanding to Intervention Strategies

The growing evidence linking intestinal MNPs to local and systemic dysfunction raises an important question: How can these effects be mitigated under realistic exposure conditions? Although complete avoidance of environmental MNP exposure is currently impractical, recent studies suggest that several intervention strategies may reduce particle bioavailability, preserve gut homeostasis, or attenuate downstream organ injury. These approaches range from dietary modulation and microbiota-based therapies to engineered biological systems and emerging physicochemical technologies (Fig. 6). However, it must be explicitly emphasized that these interventions are currently confined to conceptual frameworks and preclinical animal models. There is a distinct lack of clinical evidence in human populations, and none of these approaches are currently ready for widespread therapeutic or public application.

**Fig. 6.**
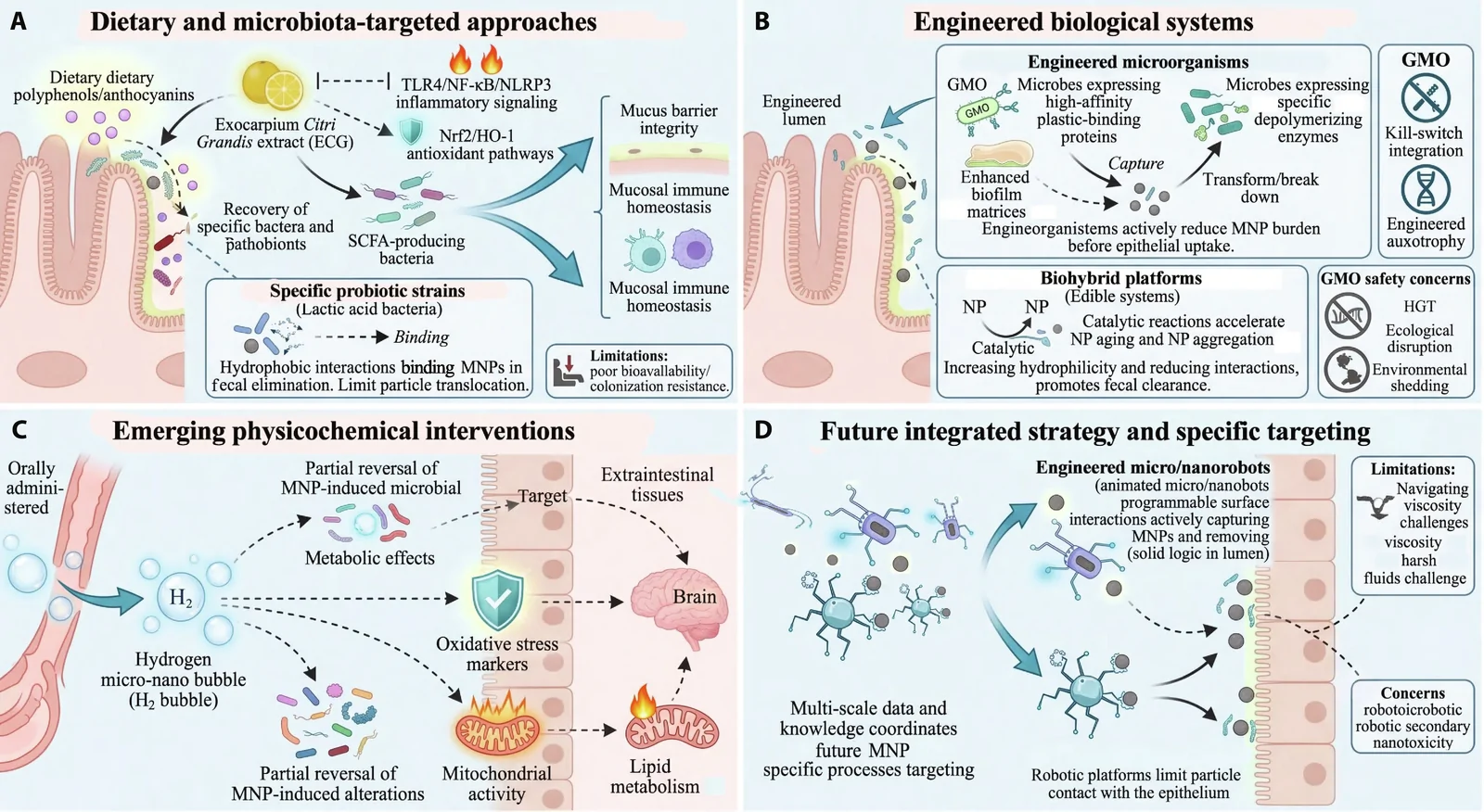
Emerging strategies and interventions to mitigate micro/nanoplastic (MNP) toxicity. (A) Dietary and microbiota-targeted approaches for intestinal protection. Schematic diagram of dietary extracts and specific probiotic strains promoting mucosal homeostasis and MNP elimination. (B) Schematic diagram of engineered biological systems used to capture and degrade MNPs via genetically modified microorganisms and biohybrid platforms. (C) Overview of emerging physicochemical interventions using hydrogen micro-nano bubbles to alleviate MNP-induced oxidative stress and metabolic alterations. (D) Diagram of future integrated strategies utilizing engineered micro/nanobots for specific MNP targeting and removal. Dashed lines denote hypothetical, proposed, or indirect pathways that require further experimental validation. ECG, Eriocaulon Cinereum Grandis extract; TLR4, Toll-like receptor 4; NF-κB, nuclear factor κB; NLRP3, NLR family pyrin domain containing 3; Nrf2, nuclear factor erythroid 2-related factor 2; HO-1, heme oxygenase-1; SCFA, short-chain fatty acid; GMO, genetically modified organism; HGT, horizontal gene transfer; NP, nanoplastic; MNP, micro/nanoplastic.

### Dietary and microbiota-targeted approaches for reducing intestinal MNP burden

Among currently available interventions, dietary strategies are the most readily applicable at the population level. Increasing evidence suggests that the intestinal microbiota is a central determinant of MNP toxicity, making microbial preservation an attractive target for intervention [59]. Experimental and early clinical studies indicate that polyphenol-rich dietary supplements can partially counteract MNP-induced microbial dysbiosis and immune imbalance [159]. These compounds appear to support the recovery of SCFA-producing bacteria while suppressing opportunistic pathobionts, thereby helping to maintain mucus barrier integrity and mucosal immune homeostasis [160,161]. Anthocyanins and other plant-derived bioactive molecules may exert similar effects by modulating microbial composition and reducing oxidative stress [162,163]. Oral administration of Exocarpium Citri Grandis extract (ECG) appears to mitigate polystyrene MP-induced hepatointestinal injury by targeting the gut–liver axis (Fig. 6A). Mechanistically, ECG inhibits *TLR4*/*NF-κB*/*NLRP3*-mediated inflammatory signaling, activates the *Nrf2*/HO-1 antioxidant pathway, preserves intestinal barrier function, restores gut microbial homeostasis, and remodels microbiota-derived metabolism [164]. Although most available evidence comes from animal models, these findings suggest that dietary modulation could represent a practical strategy for limiting chronic low-dose MNP-associated intestinal dysfunction.

Microbiota-directed interventions may provide a more targeted approach. Several probiotic strains, particularly lactic acid bacteria (Fig. 6A), have demonstrated the capacity to bind polystyrene MNPs through hydrophobic interactions on the bacterial cell surface [165,166]. Experimental studies indicate that these microorganisms can physically adsorb MNPs within the intestinal lumen and promote fecal elimination (Fig. 6A), thereby reducing epithelial contact and limiting particle translocation [167]. While these observations remain largely preclinical, they highlight the possibility of using microbial communities not only as mediators of toxicity but also as active components of detoxification. Translating these dietary and probiotic strategies to humans faces significant practical limitations. Plant-derived polyphenols and anthocyanins notoriously suffer from poor in vivo bioavailability and rapid metabolism in the human gut (Fig. 6A), meaning the high concentrations effective in in vitro or murine models are rarely achievable systemically in humans [168,169]. Similarly, the efficacy of probiotic interventions is severely hindered by human gut colonization resistance, strain-specific variability, and the fundamental ecological differences between the murine and human microbiomes [170,171].

### Engineered biological systems for active MNP sequestration and detoxification

The emergence of synthetic biology has expanded the scope of microbiota-based interventions beyond conventional probiotics. Engineered microorganisms can be designed to express high-affinity plastic-binding proteins, enhanced biofilm matrices, or specific depolymerizing enzymes capable of capturing or transforming MNPs within the gastrointestinal tract [172]. Rather than simply restoring microbial balance, these systems aim to actively reduce intestinal particle burden before epithelial uptake occurs (Fig. 6B). Recent studies have also explored biohybrid platforms that combine living microorganisms with functional materials. One example involves edible systems capable of inducing localized catalytic reactions within the intestinal lumen to accelerate NP aging and aggregation. By increasing particle hydrophilicity and reducing epithelial interactions, these platforms may facilitate particle clearance through the feces [173].

Despite their promise, engineered microbial therapies are strictly in the conceptual stage and face formidable translational barriers. Beyond the immense regulatory and ethical hurdles of deploying genetically modified organisms (GMOs) in human populations, introducing these microbes into a densely populated human intestinal ecosystem raises severe safety concerns regarding horizontal gene transfer, irreversible ecological disruption, and environmental dissemination following fecal shedding [174,175]. Future development will therefore require robust biocontainment strategies, including chromosomal integration of synthetic circuits, engineered auxotrophy, and inducible kill-switch systems that restrict microbial survival outside the intended host environment (Fig. 6B).

### Emerging physicochemical interventions targeting downstream toxicity

Not all interventions seek to prevent particle uptake directly. An alternative strategy focuses on mitigating the biological consequences that follow MNP exposure. Among the approaches currently under investigation, hydrogen micro-nano bubble technology has attracted attention because of its antioxidant and metabolic regulatory properties. Experimental studies suggest that orally administered hydrogen micro-nano bubbles may partially reverse MNP-induced alterations in gut microbial composition and metabolic function [176]. Improvements in oxidative stress markers, mitochondrial activity, and lipid metabolism have also been reported in animal models (Fig. 6C). Although the precise mechanisms remain under investigation, these findings indicate that modulation of downstream oxidative and metabolic pathways could complement interventions aimed at reducing particle exposure itself [177]. Another emerging strategy has shown that engineered micro/nanorobots are capable of actively capturing and removing MNPs within the gastrointestinal tract through programmable surface interactions and external guidance, thereby limiting particle contact with the intestinal epithelium [178]. Although this approach represents a fascinating proof-of-concept for source-oriented intervention, it is entirely restricted to the early experimental stage (Fig. 6D). Its practical implementation faces profound limitations, including the immense challenge of navigating and retaining stability within the complex, highly viscous, and chemically harsh human gastrointestinal fluids [179]. Most critically, the introduction of engineered micro/nanorobots raises paradoxically similar concerns regarding the secondary nanotoxicity, biodistribution, and long-term clearance of the very nanomaterials used to construct these detoxification platforms [180].

Additional physicochemical approaches may emerge as understanding of MNP toxicology improves. As more mechanistic pathways are identified, particularly those involving microbiota-derived metabolites and gut–organ communication networks, intervention strategies can increasingly target specific biological processes rather than relying solely on reducing environmental exposure [181].

## Current Challenges and Outlook

Despite substantial progress in elucidating the intestinal toxicity of MNPs, several challenges continue to limit the translation of experimental findings into reliable human health risk assessment and effective intervention strategies [182]. A major limitation is the gap between experimental exposure conditions and real-world human exposure [7]. Most mechanistic studies employ relatively high particle concentrations and short exposure periods to generate measurable biological responses within practical experimental timeframes. In contrast, human exposure is typically chronic, occurs at substantially lower concentrations, and involves complex mixtures of particles originating from food, water, air, and consumer products [21,183]. As a result, biological responses observed under laboratory conditions may not fully reflect the adaptive processes that develop during long-term environmental exposure. A core challenge in MNP toxicology lies in the uncertainty of extrapolating high-dose experimental findings to environmentally relevant exposures, primarily due to the inherently nonlinear nature of biological dose–response relationships. It cannot be assumed that health risks observed under milligram-scale MNP exposures can be linearly projected to the chronic, low-dose conditions experienced by humans. Biological systems often exhibit hormetic responses, characterized by biphasic dose–response patterns in which low-level MNP exposure may activate adaptive and compensatory mechanisms, such as transient antioxidant enhancement, metabolic adjustment, or mild immune preconditioning, rather than inducing the overt pathological effects observed at higher doses [184–186]. Therefore, conventional risk assessments based solely on high-dose toxicity experiments may substantially overestimate or misrepresent the actual health consequences of environmental MNP exposure. Addressing this knowledge gap requires precisely calibrated investigations that capture environmentally realistic concentrations, long-term exposure dynamics, and tissue-specific responses. Furthermore, the health burden of MNPs cannot be fully understood by examining them as isolated stressors. In real-world scenarios, MNP exposure occurs within complex environmental mixtures and interacts with other ubiquitous risk factors, including Western dietary patterns, industrial pollutants, and tobacco-related toxicants [187,188]. An exposome-based framework that integrates cumulative, multi-stressor exposures is therefore essential to accurately elucidate how chronic MNP exposure interacts with broader environmental pressures to contribute to the increasing global prevalence of NCDs.

The considerable heterogeneity of environmental MNPs further complicates risk evaluation. Toxicity is influenced by multiple interacting characteristics, including particle size, morphology, surface chemistry, polymer composition, weathering status, and adsorbed contaminants [189]. Current mechanistic knowledge remains heavily derived from studies using commercially available polystyrene particles because of their experimental convenience and reproducibility [27]. However, evidence summarized throughout this review indicates that different polymers may induce toxicity through partly distinct biological pathways. NPs are more likely to trigger cellular internalization and organelle dysfunction, whereas MPs predominantly affect epithelial integrity, mucosal immunity, and microbiota structure [190]. Biodegradable polymers introduce an additional layer of complexity because degradation products generated within the gastrointestinal tract can actively participate in host–microbiota metabolic interactions. Recent comprehensive reviews highlight that the ingestion and biotransformation of PLA and PBAT MNPs—which result in fragmentation, soluble intermediate release, and competitive biomolecular interactions—induce significant oxidative stress, metabolic disturbances, and gut microbiota alterations across various biological models [191–193]. Consequently, findings from PLA- and PBAT-based materials suggest that environmental degradability does not necessarily predict biological safety, highlighting the need to evaluate biodegradability and toxicological outcomes as separate endpoints [69,191]. Another important challenge is the limited availability of human evidence. Although MNPs have been detected in human feces, blood, placenta, and several tissues, reported concentrations vary substantially among studies [194,195]. Differences in sampling procedures, contamination control, particle isolation methods, and analytical platforms remain major sources of uncertainty. Consequently, it is often difficult to compare datasets across studies or establish clear exposure–response relationships. Standardizing detection and quantification protocols requires urgent consensus across 3 specific dimensions: first, the development of matrix-matched, weathered reference materials to replace pristine commercial standards; second, the harmonization of tissue extraction and digestion methods (such as enzymatic versus chemical degradation) to ensure that they do not artifactually alter polymer chemistry or morphology; and third, the establishment of unified reporting metrics that mandate the inclusion of both particle number and mass concentration, strictly bounded by transparent size detection limits. The dynamic nature of the gut ecosystem presents an additional unresolved question. Most studies characterize biological responses during relatively early stages of exposure [59], whereas little is known about how the intestinal microbiota adapts to continuous MNP exposure over months or years. Because microbial communities possess considerable functional redundancy and ecological resilience, early compositional disturbances may recover over time [196]. Conversely, subtle metabolic alterations that appear minor during short-term exposure may gradually accumulate and contribute to chronic disease susceptibility [197]. Distinguishing transient responses from stable functional reprogramming will be critical for understanding long-term health consequences.

Addressing these challenges will require a shift from simplified exposure models toward more realistic experimental frameworks that quantitatively align with human biomonitoring data. To achieve this, researchers must transition away from acute bolus dosing (e.g., oral gavage) of pristine monodisperse polystyrene. Realistic exposure paradigms should be designed by incorporating multi-polymer mixtures—such as utilizing distinct plastic types structured around component-dominant ratios rather than uniform concentration gradients—alongside photo-aged and contaminant-adsorbed MNP mixtures directly into animal chow or drinking water. This matrix-bound, continuous feeding approach accurately mimics the chronic, low-dose dynamics of human dietary ingestion and allows for the natural formation of gastrointestinal biomolecular coronas [7,198]. Rather than deploying advanced technologies broadly, future studies must target specific mechanistic bottlenecks. For instance, spatial transcriptomics should be utilized to precisely map the colocalization of trapped MNPs with localized epithelial stress responses and focal barrier disruption in intact tissues. Furthermore, tracking epigenetic drift through long-term longitudinal studies is necessary to determine whether chronic mucosal inflammation induces heritable metabolic vulnerabilities [199]. Finally, the development of next-generation, vascularized human organoids (cocultured with resident immune cells and a stable microbiota) is essential to decipher the specific routes by which MNPs and microbial metabolites cross the gut–vascular barrier—a central question that conventional in vitro systems cannot resolve [200]. At the same time, comparative studies across different polymer classes are needed to distinguish conserved mechanisms from polymer-specific effects. To answer the fundamental question of why this evidence changes our evaluation of MNP health risks: It dictates that we can no longer assess MNP safety solely by quantifying particle burdens in isolated organs. If the primary pathogenic driver is gut-mediated systemic disruption, then future risk assessments, regulatory frameworks, and clinical interventions must pivot. They must move beyond simple “dose-and-translocation” models to prioritize functional biomarkers of mucosal immunity, microbiome resilience, and metabolic crosstalk. Only by recognizing the gastrointestinal tract as the central biological amplifier of environmental particulate stress can we accurately chart the mechanistic path from global plastic pollution to the rising tide of chronic systemic diseases.

## Data Availability

Data will be made available on request.
